# An ART-fold Rhs toxin from *Pluralibacter gergoviae* defines Tne5, a novel family of NAD(P) glycohydrolases effectors

**DOI:** 10.1016/j.jbc.2026.113182

**Published:** 2026-05-22

**Authors:** Jonas B. Desjardins, Martin Durrmeyer, Olivier Bornet, Eric Cascales

**Affiliations:** 1 Laboratoire d’Ingénierie des Systèmes Macromoléculaires (LISM – UMR7255), Aix-Marseille Univ, CNRS, UMR7255, Marseille, France; 2 NMR Facility, Institut de Microbiologie de la Méditerranée (IMM – UAR2044), Aix-Marseille Univ, CNRS, Marseille, France

**Keywords:** ADP-ribosyltransferase, NADase, NAD(P) glycohydrolase, *Pluralibacter gergoviae*, polymorphic toxin, Rhs, toxin–immunity pair, type VI secretion

## Abstract

The type VI secretion system (T6SS) is a widespread bacterial nanomachine that mediates interbacterial competition by delivering toxic effectors into neighboring cells. Among these, enzymes targeting nicotinamide adenine dinucleotide (NAD) cofactors and NAD phosphate (NAD(P)) are particularly potent because they rapidly disrupt redox homeostasis and central metabolism. Several families of T6SS-associated NAD(P)-consuming effectors (Tne1-Tne4) have been described. Here, we characterize a T6SS-associated Rearrangement hot spot toxin from *Pluralibacter gergoviae*. Competition assays show that *P. gergoviae* kills *Escherichia coli* in a T6SS-dependent manner. Heterologous production reveals that the Rearrangement hot spot C-terminal extension is toxic in the *Escherichiacoli* cytoplasm and that co-production with the protein encoded downstream neutralizes this activity. AlphaFold3 modeling predicts that the toxin adopts an ADP-ribosyltransferase-like α/β fold with a putative catalytic pocket accommodating NADH. By contrast to T6SS ART toxins described so far, the toxin does not inhibit transcription or translation but instead depletes NAD(P) *in vitro* and causes intracellular NAD depletion in intoxicated cells. Nuclear magnetic resonance analyses further show that the toxin hydrolyzes NAD ^+^ into nicotinamide and ADP-ribose. Phylogenetic analyses and structural modeling show that this effector defines a new ART-related family of NAD(P) glycohydrolases broadly distributed across antagonistic systems, which we propose to name Tne5.

Bacteria deploy antibacterial weapons to compete for nutrients and space in densely populated niches (1, 2, 3, 4, 5). One of the key actors of bacterial competition is the type VI secretion system (T6SS), a sophisticated nanomachine belonging to the broad family of contractile injection systems, which uses a contractile mechanism to deliver effectors into neighboring cells (6, 7, 8, 9). The activity of the T6SS impacts the composition and organization of microbial communities, provides a privileged access to nutrients, and promotes colonization of the niche (4, 10, 11, 12, 13). The T6SS is comprised of a membrane complex that anchors a cytoplasmic phage-related structure made of an assembly platform, the baseplate, and of the tail tube/sheath complex (6, 7, 8, 14). The tail tube/sheath complex is composed of a needle wrapped by the contractile sheath, whose contraction propels the needle towards target cells (7, 15). The needle is made of an inner tube of stacked Hcp hexamers tipped by a VgrG- Proline-Alanine-Alanine-Arginine (PAAR) spike complex (7, 8, 15). The effectors are either independent polypeptides that associate, directly or through the help of adaptors and chaperones, to the needle, or domain fused to structural subunits of the needle, Hcp, VgrG or PAAR (9, 16, 17, 18, 19). These latter effectors belong to the family of polymorphic toxins (PTs), which are comprised of a C-terminal effector domain fused to an N-terminal trafficking domain that targets the toxin to the proper delivery apparatus (20). Beyond these trafficking and toxic domains, PTs may contain supplementary structural elements. For example, T6SS-associated PAAR PTs commonly possess transmembrane helices that are proposed to promote insertion into target cell membranes, or Rearrangement hot spot (Rhs) domains that form cocoons encapsulating the toxic C-terminal extension (21, 22, 23, 24, 25, 26, 27).

Antibacterial T6SS effectors target essential molecules, macromolecules and processes of the bacterial physiology: cell wall integrity, membrane homeostasis, nucleic acids, cofactors and nucleotides, or the translation machinery (9, 19, 28, 29, 30). Within this diversity, a broad family of T6SS enzymatic effectors is related to nicotinamide adenine dinucleotide (NAD) or NAD phosphate (NADP) (31). This family includes ADP-ribosyltransferases (ART) that catalyze the transfer of ADP-ribose from NAD to proteins or nucleic acids (32, 33, 34, 35, 36, 37, 38, 39, 40, 41). Structurally, ARTs fold as a split β-sheet typically composed of six strands in the order β4-β5-β2/β1-β3-β6, presenting diverse catalytic triads (H-H-h, H-Y-[QED], R-[ST] or R-H-h) that define major lineages (32, 33, 36). In addition, other highly potent effectors with ART-like folds can catalyze NAD or NADP (NAD(P)) degradation, such as NAD(P) glycohydrolases (NADases), or cyclization reactions, such as ADP-ribosyl cyclases (ARC) (42, 43, 44, 45, 46, 47). These NADase or ARC activities rapidly compromise redox balance and metabolism by depleting NAD(P) at high rates, leading to growth arrest or death (31). Recent comparative analyses have classified T6SS-associated NAD(P) glycohydrolases into four major families (Type VI secretion NADase effectors 1–4, Tne1-Tne4) based on phylogenetic analyses (42, 43, 45).

Here, we show that *Pluralibacter gergoviae* ATCC33028 (formerly *Enterobacter gergoviae*) carries a T6SS gene cluster encoding a putative polymorphic toxin comprising PAAR and Rhs domains, Rhs^Pg^. Competition assays demonstrate that the *P. gergoviae* T6SS is active and eliminates *Escherichia coli*. Heterologous expression in *E. coli* shows that the Rhs^Pg^ C-terminal extension (Rhs^Pg^-CT) is toxic and can be neutralized by the protein encoded downstream of *rhs*. Rhs^Pg^-CT AlphaFold3 structural modeling predicts that the domain adopts an ART-like α/β fold with a putative catalytic cleft accommodating NAD, comprising residues Lys1392, and Glu1482. Mutational analysis further shows that Lys1392 is important for toxicity, while Glu1482 is not. Functional assays demonstrate that Rhs^Pg^-CT does not inhibit transcription or translation and does not ADP-ribosylate target cell proteins but rather depletes NAD *in vivo* and hydrolyzes NAD^+^ onto nicotinamide and ADP-ribose *in vitro*, classifying it as a member of the Tne family. Phylogenetic analyses further show this Tne defines a new family of NAD(P) glycohydrolases, which we term Tne5, shedding light on the diversification of ART-like folds in bacterial competition.

## Results

### A T6SS locus in *Pluralibacter gergoviae* mediates antibacterial activity

**Figure 1:**
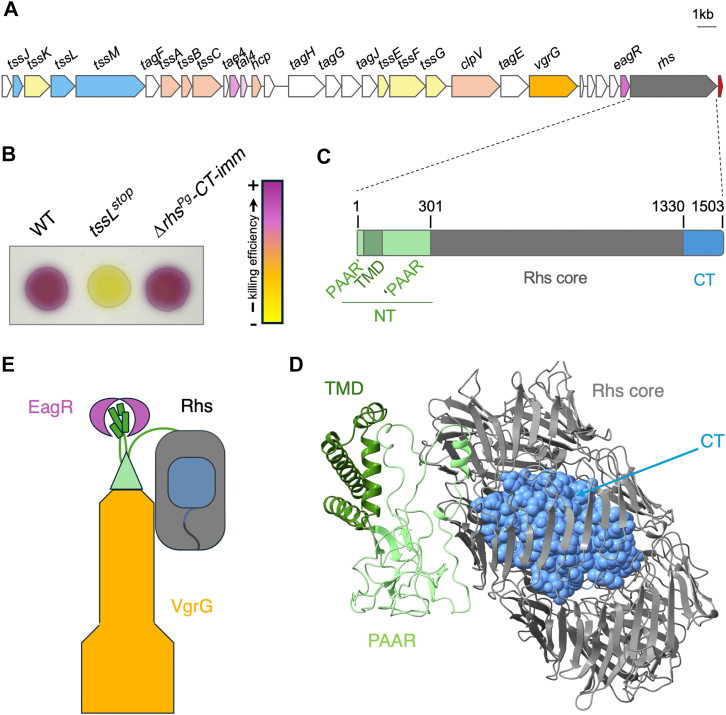
**A T6SS locus in *Pluralibacter gergoviae* encodes a putative Rhs toxin-immunity pair and mediates antibacterial activity.***A*, schematic representation of the *P. gergoviae* ATCC33028 T6SS gene cluster. The different *tss* (*blue*, *membrane complex; yellow*, *baseplate; flesh*, *tail tube/sheath*) and *tag* (*white*) genes are indicated, as well the genes encoding a VgrG spike (*orange*), a predicted Tae4-Tai4 pair (*pink*), a EagR chaperone (*purple*), a Rhs protein (*gray*) and its putative immunity (*red*). *B*, interbacterial competition assay. WT *P. gergoviae* or its isogenic *tssL*^stop^ and Δ*rhsPg-CT-imm* mutants were mixed with *lacZ*^*+*^*E. coli* recipients, spotted on LB agar plates and incubated for 4 h at 37 °C. Recipient cell lysis after competition was revealed using CPRG (*yellow*), which turns *purple* upon hydrolysis by β-galactosidase from lysed *lacZ*^*+*^ recipient cells. The killing efficiency heatmap (from *yellow* (no killing) to *purple* (killing)) is shown on the *right*. *C* and *D*, schematic representation (*C*) and AlphaFold3 structural model (*D*) of the Rhs^Pg^ protein, highlighting the different domains and their boundaries: PAAR domain (PAAR’ or prePAAR and ‘PAAR in *light green*), trans-membrane domain (TMD in *dark green*), Rhs core (*gray*), and C-terminal extension (*blue*). The confidence scores, pLDDT and PAE are presented in Fig. S1. *E*, schematic representation of the Rhs^Pg^ effector (same colors as panels *C* and *D*) loaded to the VgrG spike (*orange*). A dimer of EagR chaperones (*purple*) is shown bound to the Rhs^Pg^ TMD. The AlphaFold3 model of the VgrG-Rhs-EagR complex, and its confidence scores, pLDDT and PAE are presented in Fig. S2. LB, Lysogeny Broth; Rhs^Pg^-CT, Rhs^Pg^ C-terminal extension; Rhs, rearrangement hot spot.

*Pluralibacter gergoviae* is an environmental Enterobacterales species that can occasionally cause opportunistic urinary tract infections (48). Because of its high tolerance to widely used preservatives such as parabens and isothiazolinones (49, 50, 51), *P. gergoviae* has been associated with multiple contamination outbreaks and recalls of cosmetic, personal care and hygiene products (52, 53, 54). Genome screening of *P. gergoviae* ATCC33028 revealed a T6SS gene cluster containing the core genes (*tssA-M*), accessory modules (*tag* genes), and toxin-immunity pairs such as a putative Tae4 amidase, consistent with a functional antibacterial system (Fig. 1*A*). Indeed, antibacterial competition assays using the degradation of chlorophenol-red β-D-galactopyranoside (CPRG, yellow), a membrane-impermeable chromogenic substrate of the β-galactosidase, into chlorophenol-red by the β-galactosidase released by the lysis of *lacZ*^*+*^ recipient cells, as a proxy, demonstrated that WT *P. gergoviae* eliminated *E. coli* K12 W3110 whereas its isogenic *tssL* mutant did not (Fig. 1*B*), demonstrating T6SS-dependent antibacterial activity.

### The *P. gergoviae* T6SS encodes a rhs polymorphic toxin

The *P. gergoviae* T6SS gene cluster carries a *rhs* gene (Rhs^Pg^, NCBI Reference Sequence: WP_330993187; GeneBank Identifier: AVR02352; locus tag: A8H26_06345) immediately followed by a small gene encoding a predicted cytoplasmic protein (GeneBank Identifier: AVR02353; locus tag: A8H26_06350), a gene organization typical of Rhs-immunity pairs. Domain annotation of the 1503-amino-acid (aa) Rhs^Pg^ protein demonstrated that it shares the canonical modular organization of T6SS Rhs toxins (24, 25, 26, 27): (*i*) an N-terminal region (amino-acids (aa) 1 to 301) comprising a PAAR domain (PAAR’, aa 1–15 and ‘PAAR, aa 98–301) that likely specifies transport by the T6SS through association with the VgrG spike protein (55, 56, 57) and a predicted 4-helix transmembrane domain (TMD, aa 16–97), in agreement with the presence of a gene upstream *rhs* encoding a putative TMD-protecting chaperone EagR (21, 22), (*ii*) a central YD-repeat Rhs encapsulation shell (aa 302–1329), and (*iii*) a C-terminal extension (aa 1330–1503, Rhs^Pg^-CT) that typically carries the toxic activity (Fig. 1, *C* and *D*). These three regions are separated by two cleavage sites that are responsible for the release of the buried C-terminal extension: the N-terminal PVHAATGV motif resembling the PVSMVTGE and PVYVASGE signatures of *Aeromonas dhakensis* and *Photorhabdus laumondii* Rhs proteins (24, 58), and the canonical autoproteolysis DPhGL-X_17_-DPhGL C-terminal motif (24, 58). AlphaFold3 modeling prediction confirmed the canonical structural organization of Rhs proteins, with the C-terminal extension encapsulated within the Rhs core shell (Figs. 1*D* and S1). This prediction also suggested that the N-terminal domain has a typical PAAR fold, that potentially binds to the tip of the VgrG protein encoded six genes upstream of *rhs* (Fig. S2). Finally, as experimentally demonstrated for the *Pseudomonas aeruginosa* Tse6 effector (21, 22), the two EagR subunits are predicted to form a horseshoe-shaped protecting shell around the putative TMD (Fig. S2). Overall, this organisation suggests that the Rhs polymorphic toxin uses its PAAR domain to bind to VgrG for its translocation, with the EagR chaperones required for maintaining the TMD in a state competent for translocation (Fig. 1*E*).

**Figure 2:**
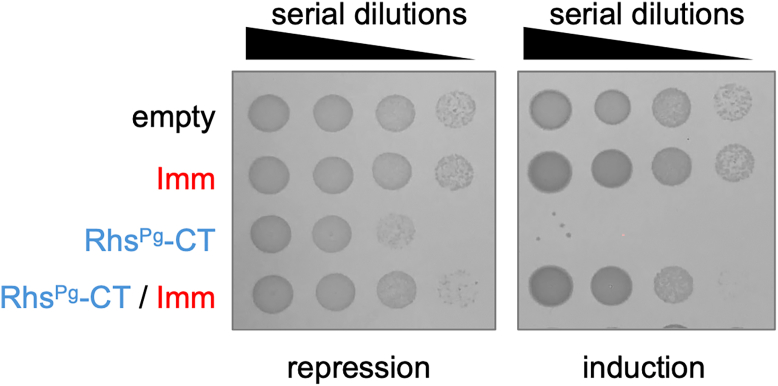
**The Rhs^Pg^-CT is toxic in the *E. coli* cytoplasm and is neutralized by the downstream immunity protein.** Toxicity assay in the heterologous host *E. coli.* Overnight cultures of *E. coli* cells producing the Rhs^Pg^-CT from the low-copy vector pNDM220, the putative immunity protein (A8H26_06350, Imm) from the pBAD33 vector, or both, were serially diluted (10^−1^ to 10^−4^) and spotted on LB agar plates supplemented with 1% of glucose (*left* panel, repression), or with 0.05 mM of IPTG and 0.2% of L-arabinose (*right* panel, induction). Empty, empty pNDM220 and pBAD33 vectors. LB, Lysogeny Broth; Rhs^Pg^-CT, Rhs^Pg^ C-terminal extension.

To test whether the Rhs^Pg^ C-terminal extension has antibacterial activity, the sequence downstream the C-terminal autoproteolysis motif (corresponding to aa 1330–1503) was cloned into the low-copy, IPTG-inducible, pNDM220 vector and heterologously produced in the *E. coli* cytoplasm. Figure 2 shows that induction of the expression of Rhs^Pg^-CT was toxic in *E. coli*. Co-expression of the A8H26_06350 gene, located downstream of *rhs*, from the pBAD33 vector, restored growth (Fig. 2), suggesting it neutralizes Rhs^Pg^-CT toxicity. Taken together, these results demonstrate that Rhs^Pg^-CT is an antibacterial toxin acting in the cytoplasm, whereas A8H26_06350 encodes its cognate immunity protein. However, Rhs^Pg^ is probably not the only antibacterial T6SS effector in *P. gergoviae* as a mutant deleted of the Rhs^Pg^-CT sequence was still capable of eliminating *E. coli* W3110 (Fig. 1*B*).

### Rhs^Pg^-CT shares a typical ART fold

**Figure 3:**
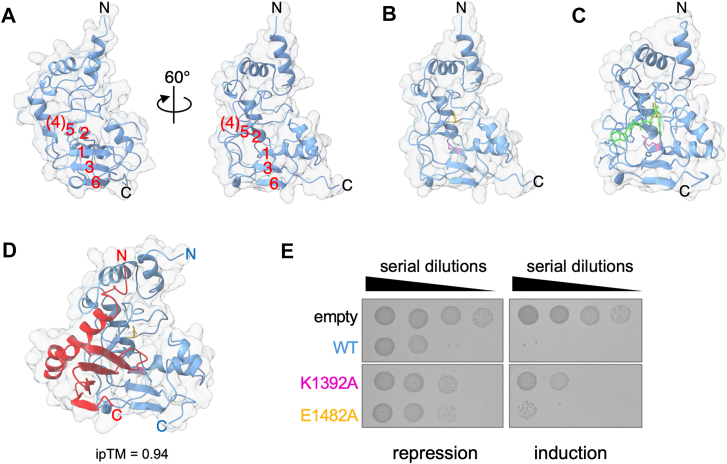
**Rhs^Pg^-CT shares an ART fold with a putative cavity accommodating the NADH and occluded by its cognate immunity.***A*, ribbon representation of the AlphaFold3 prediction model of Rhs^Pg^-CT. The β-strands of the split β-sheet are numbered in *red*. The region located at the position of β-strand β4 in the typical ART fold is indicated in brackets. The surface structure is shown in *gray* transparent. N, N-terminus; C, C-terminus. The confidence score, pLDDT and PAE are presented in Fig. S3, *A* and *B*. *B*, ribbon representation of the AlphaFold3 prediction model of Rhs^Pg^-CT highlighting the positions of residues K1392 (*pink*) and E1482 (*orange*). *C*, ribbon representation of the AlphaFold3 prediction model of Rhs^Pg^-CT bound to NADH (ipTM score = 0.96). The Rhs^Pg^-CT residues K1392 and E1482 are shown in *pink* and *orange*, whereas the NADH is shown in *green*. The confidence score, pLDDT and PAE are presented in Fig. S3, *C* and *D*. *D*, ribbon representations of the AlphaFold3 prediction model of the Rhs^Pg^-CT/Imm complex (ipTM score = 0.94). The toxin domain is shown in *blue* whereas the immunity is shown in *red*. The surface structure is shown in *gray* transparent. The confidence score, pLDDT and PAE are presented in Fig. S3, *E* and *F*. *E*, toxicity assay in the heterologous host *E. coli.* Overnight cultures of *E. coli* cells producing WT Rhs^Pg^-CT or the K1392A or E1482A variants from the low-copy vector pNDM220 were serially diluted (10^−1^ to 10^−4^) and spotted on LB agar plates, supplemented with 1% of glucose (*left* panel, repression), or with 0.05 mM of IPTG (*right* panel, induction). Empty, empty pNDM220 vector. LB, Lysogeny Broth; Rhs^Pg^-CT, Rhs^Pg^ C-terminal extension.

AlphaFold3 modeling of Rhs^Pg^-CT revealed an ART-like fold, comprising a compact α/β catalytic scaffold built around a split β-sheet delimiting a cleft and flanked by 5 α-helices (Figs. 3*A* and S3, *A* and *B*). As in typical ARTs, the β-sheet core is composed of six β-strands organized as two perpendicular 3-stranded β-sheets in the order β4-β5-β2/β1-β3-β6 (Fig. 3*A*). However, β-strand β4 is missing or not predicted by AlphaFold3 in Rhs^Pg^-CT (Fig. 3*A*). In ART enzymes, NAD^+^ positioning and catalysis rely on a triad of residues usually located on strands β1, β2 and β5, defining major families (H-Y-E, R-S-E, and h-h-H) (32). Sequence analyses of Rhs^Pg^-CT suggested that Lys1392 and Glu1482 may constitute the putative catalytic pocket (Fig. 3*B*). A highly confident model including NADH (ipTM = 0.96) positioned the dinucleotide in the Rhs^Pg^-CT cleft (Figs. 3*C* and S3, *C* and *D*) whereas the AlphaFold3 co-model of Rhs^Pg^-CT with its immunity protein (ipTM = 0.94) predicted formation of a tight complex between the two partners, in which a loop of the immunity penetrates deeply into the Rhs^Pg^-CT cleft, sterically occluding the putative NAD-binding pocket (Figs. 3*D* and S3, *E* and *F*). Alanine substitution of Rhs^Pg^-CT residue Glu1482 had a very weak reduction in activity, whereas the K1392A mutation significantly impaired toxicity (Fig. 3*E*).

### Rhs^Pg^-CT does not inhibit transcription or translation, and does not detectably ADP-ribosylate proteins

The structural model therefore suggests a dinucleotide-binding ART-like scaffold but does not by itself distinguish between ADP-ribose transfer and dinucleotide hydrolysis activities. We thus tested both ADP-ribosylation and NAD(P) consumption. Given that most T6SS-associated ART-fold toxins have been shown to inhibit translation in a NAD-dependent manner, through the modification of RNAs or translation factors (38, 39, 40), we first asked whether Rhs^Pg^-CT impacts protein synthesis using a coupled *in vitro* transcription-translation (IVT) assay. Rhs^Pg^-CT was first synthesized by IVT in the absence of NAD and then incubated in a new IVT reaction in the presence of a DNA template encoding GFP. Fig. S4*A* shows that Rhs^Pg^-CT did not prevent GFP production in the presence or absence of NAD^+^, demonstrating that Rhs^Pg^-CT does not inhibit transcription and translation. To identify potential protein targets of Rhs^Pg^-CT, we incubated IVT-synthesized Rhs^Pg^-CT with *E. coli* cell lysates in the presence of 6-biotin-17-NAD^+^, a NAD^+^ derivative in which the ADP-ribose moiety is decorated with a biotin. The biotinylated protein profiles did not differ detectably in the absence or presence of Rhs^Pg^-CT (Fig. S4*B*), suggesting that Rhs^Pg^-CT does not ADP-ribosylate cellular proteins.

### Rhs^Pg^-CT has NAD(P)^+^ glycohydrolase activity

**Figure 4:**
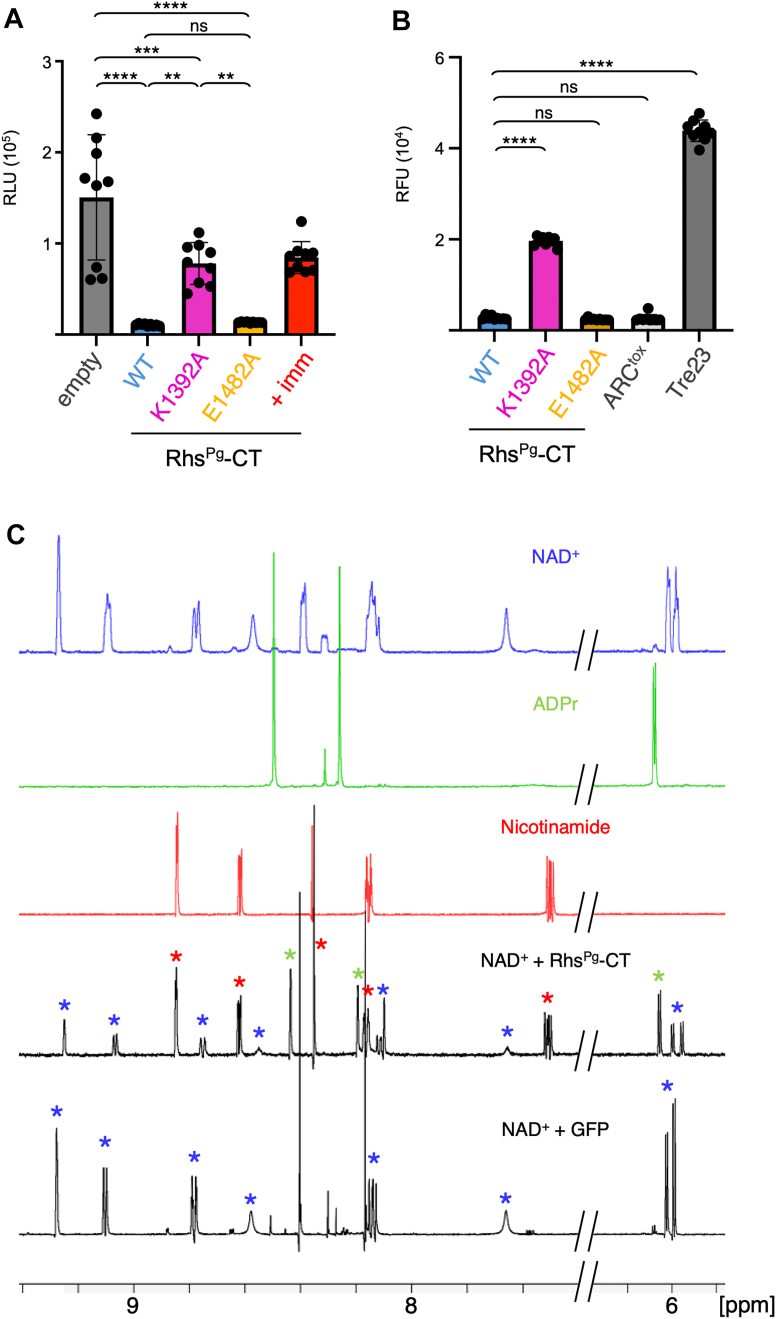
**Rhs^Pg^ hydrolyzes NAD *in vivo* and *in vitro* and releases nicotinamide and ADP-ribose.***A*, i*n vivo* NAD glycohydrolase assay. Luminescence-based quantification of intracellular NAD levels in *E. coli* cells carrying the empty vector (*gray bar*), or producing WT, *blue bar* Rhs^Pg^-CT, or its K1392A (*purple bar*) or E1482A (*orange bar*) variants, or WT Rhs^Pg^-CT together with its cognate immunity protein (Imm, *red bar*). RLU, relative luminescence units. *Bars* represent the mean of technical triplicates from three independent cultures (each independent value indicated by *circle*s). Standard deviations are indicated. The stars denote statistically significant differences between conditions (one-way ANOVA; ∗∗, *p* < 0.01; ∗∗∗, *p* < 0.001; ∗∗∗∗, *p* < 0.0001; ns, non-significant). *B*, i*n vitro* NAD^+^ glycohydrolase assay. Fluorescence-based assay measuring NAD^+^ levels after incubation of 0.6 mM of NAD^+^ with 0.07 μM of WT Rhs^Pg^-CT (*blue bar*) or its K1392A (*purple bar*) or E1482A (*orange bar*) variants for 30 min. Control reactions with 0.07 μM of ARC^tox^ (NAD(P)-consuming, *light gray bar*) or of Tre23 (ART, dark *gray bar*) were included. RFU, relative fluorescence units. *Bars* represent the mean of nine independent reactions (each independent value indicated by *circles*). Standard deviations are indicated. ∗∗∗∗ denotes a statistically significant difference between conditions (one-way ANOVA; *p* < 0.0001; ns, non-significant). *In vitro* NADPH degradation assays are shown in Fig. S5. *C*, nuclear magnetic resonance spectroscopy analyses. Enlargement of the aromatic region of the one-dimensional ^1^H Nuclear magnetic resonance spectra of reference compounds (NAD^+^, *blue*; ADP-ribose, *green*; nicotinamide, *red*), and the reaction mixtures obtained after 30-min incubation of NAD^+^ in the presence of 0.07 μm of GFP or of Rhs^Pg^-CT, recorded at 600 MHz. In the NAD^+^+GFP and NAD^+^ + Rhs^Pg^-CT spectra, the asterisks indicate the resonances corresponding to the NAD^+^ (*blue asterisk*), the ADP-ribose (*green asterisk*) and the nicotinamide (*red asterisk*). The entire spectra are shown in Fig. S6. Rhs^Pg^-CT, Rhs^Pg^ C-terminal extension.

The previous data suggested that if Rhs^Pg^-CT engages NAD, it may preferentially catalyze NAD hydrolysis rather than ADP-ribose transfer to protein targets. Indeed, DALI searches of the Rhs^Pg^-CT AlphaFold3 model against the Protein Data Bank (PDB) returned the *Streptococcus pyogenes* β-NAD^+^ glycohydrolase (PDB 4KT6, RMSD 2.6 Å, Z-score 13.1) as the closest structural homologue. We therefore asked whether production of the toxin in *E. coli* cells affects the intracellular NAD pools. Using a luminescence-based assay, we found that production of WT Rhs^Pg^-CT caused a collapse of cellular NAD levels, whereas co-production of the cognate immunity protein prevented NAD depletion (Fig. 4*A*). Consistent with the toxicity assays, the K1392A variant exhibited reduced activity, while the E1482A substitution had little or no effect (Fig. 4*A*). Thus, Rhs^Pg^-CT intoxication causes depletion of intracellular NAD *in vivo*, providing support to a NAD glycohydrolase activity. To further test this, we quantified NADase activity *in vitro* using a fluorescence-based assay. NAD^+^ or NADPH levels were measured after 30 min of incubation with Rhs^Pg^-CT obtained from IVT. Rhs^Pg^-CT strongly depleted both NAD^+^ and NADPH, at levels comparable to the *Pantoea ananatis* ARC ADP-ribosyl cyclase (ARC^tox^, (47)), whereas the *P. laumondii* ART Tre23 toxin had no measurable effect (Figs. 4*B* and S5). As observed *in vivo*, the Rhs^Pg^-CT K1392A variant yielded an intermediate phenotype with partial depletion of the NAD(P)H pool while the E1482A mutation had little or no impact (Figs. 4*B* and S5). To get further information on the reaction catalyzed by Rhs^Pg^-CT, we identified the reaction products of the *in vitro* NAD^+^ degradation assay by nuclear magnetic resonance (NMR) spectroscopy. In the presence of Rhs^Pg^-CT, characteristics NAD^+^ signals (6.0, 6.02, 8.15, 8.4, 9.1 and 9.3 ppm) strongly decreased and peaks corresponding to nicotinamide (NAM; 7.5, 8.15, 8.6 and 8.8 ppm) and ADP-ribose (5.1, 5.2 and 6.1 ppm) appeared, whereas control reactions containing GFP did not show modification of the NAD^+^ signals (Figs. 4*C* and S6). Taken together, these results demonstrate that the *P. gergoviae* Rhs C-terminal domain hydrolyzes NAD^+^ into NAM and ADP-ribose.

### Phylogenetic analyses define a new ART-related NADase clade, Tne5

**Figure 5:**
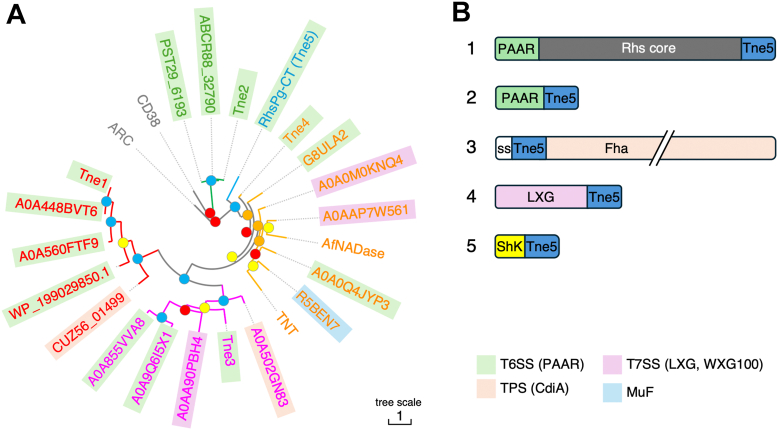
**Phylogenetic and genomic analyses define Tne5 as a new family of ART-related NAD(P) glycohydrolases.***A*, maximum-likelihood phylogenetic tree of prototypical and representative members of the Tne1 (*red lettering*), Tne2 (*green lettering*), Tne3 (*pink lettering*) and Tne4 (*orange lettering*) NADase families, canonical NAD(P) glycohydrolases (Tuberculosis Necrotizing Toxin, AfNADase), ADP-ribosyl cyclases (ARC^tox^ and human CD38), and Rhs^Pg^-CT. Toxin associated with T6SS, contact-dependent inhibition, type VII secretion systems and MuF systems are underlined in *green*, flesh, *pink* and *blue*, respectively. Branch support values are indicated by *colored circles* at nodes, corresponding to ultrafast bootstrap values (*blue*, >95; *yellow*, 80–90; *orange*, 70–80; *red*, <70). Rhs^Pg^-CT clusters in a distinct clade, separate from the previously defined Tne1-Tne4 families. *B*, representative genomic contexts of Tne5 domains identified by JackHMMER searches. Tne5 domains occur in five major architectures, as C-terminal extensions of polymorphic toxins associated with T6SS-dependent PAAR (*green*) and Rhs (*gray*) domains, contact-dependent growth inhibition (flesch), type VII secretion systems -dependent LXG domains (*pin*k), or ShK elements (*yellow*). Rhs, rearrangement hot spot; Rhs^Pg^-CT, Rhs^Pg^ C-terminal extension.

To position Rhs^Pg^-CT within the tree of T6SS-associated NAD(P)-consuming effectors, we conducted phylogenetic analyses. Maximum-likelihood trees built from representative members of the four previously defined Tne families (Tne1-Tne4) as well as characterized NAD-consuming toxins (*Mycobacterium tuberculosis* Tuberculosis Necrotizing Toxin (59)), *Aspergillus fumigatus* AfNADase (60), and *P. ananatis* ARC^tox^ (47)) indicate that Rhs^Pg^-CT does not cluster with any of these groups but instead forms a distinct clade (Fig. 5*A*). Although branch support values vary across the tree, this analysis suggests that Rhs^Pg^-CT defines a new family, which we designate Tne5, and its cognate immunity protein Tni5.

To assess the distribution of Tne5-like effectors, we performed iterative homology searches using JackHMMER with the *P. gergoviae* Rhs^Pg^ Tne5 sequence as a query. This analysis identified bacterial Tne5 homologues associated with T6SS PAAR or PAAR-Rhs domains, and contact-dependent inhibition systems, and type VII secretion systems signatures, as well as more distantly related Tne5-like domains fused to ShK modules in nematodes and sea anemones (Fig. 5*B*).

## Discussion

In this study, we demonstrated that *Pluralibacter gergoviae* encodes a T6SS gene cluster, bearing all the core genes, essential for the assembly of a functional T6SS (Fig. 1*A*). This T6SS is active in laboratory conditions and displays antibacterial activity against *E. coli* cells (Fig. 1*B*). Genomic analyses suggest that this T6SS gene cluster carries two toxin-immunity pairs, including a putative Tae4 amidase and its cognate immunity Tai4, and a Rhs polymorphic toxin followed by a small open reading frame encoding a cytosolic protein (Fig. 1*A*). This Rhs protein is predicted to comprise a PAAR domain followed by a bundle of transmembrane helices, a Rhs core and a C-terminal extension (Fig. 1, *C* and *D*). This organization is typical of Rhs polymorphic toxins delivered by the T6SS, in which the PAAR domain specifies association to the VgrG spike protein (56, 57, 58). Indeed, AlphaFold3 modeling suggests formation of a VgrG-Rhs complex involving binding of the PAAR domain to the VgrG tip (Figs. 1*E* and S2). In addition, transmembrane helices are usually protected by chaperones of the EagR family (21, 22), and such a putative chaperone is encoded upstream the *rhs* gene (Figs. 1, *A* and *E*, 2). Future biochemical studies will be required to experimentally test these interactions. Nevertheless, heterologous expression of the Rhs C-terminal extension in *E. coli* was toxic and co-expression of the downstream gene restored viability (Fig. 2), demonstrating that *rhs* and the downstream gene encode a *bona fide* polymorphic toxin-immunity pair. However, a strain deleted of the sequence encoding the Rhs^Pg^ effector domain efficiently eliminated *E. coli*, likely due to the presence of additional effector candidates such as Tae4.

While experimental validation is required, the AlphaFold3 model of the toxic C-terminal Tne5 extension revealed an (ART-like α/β fold, consisting of a split β-sheet composed of five strands in the order β5-β2/β1-β3-β6, buttressed by helices to create a putative dinucleotide-binding cleft (Fig. 3, *A*–*C*). However, the strand usually at position β4 is missing, or more likely not well predicted as an unstructured sequence is present at position of the typical β4. In the ligand-bound AlphaFold3 model (Fig. 3, *B* and *C*), the NADH molecule is accommodated within a relatively deep cavity and is stabilized by multiple side-chain interactions. The adenosine moiety is positioned through contacts with Arg1399, Pro1404, and Gln1487, whereas the ribose is stabilized by Phe1409 and Asn1467. The nicotinamide ring is located within a hydrophobic environment formed by Ile1393 and Leu1434. Lys1392 and Glu1482 are predicted to contribute a basic-acidic pocket that accommodates the pyrophosphate/ribose and nicotinamide moieties and hence may participate in NAD(P) engagement and catalysis (Fig. 3, *B* and *C*). The importance of this pocket is echoed by the Tne5-Tni5 model, which places the immunity protein over the cleft in a way that would sterically occlude access of the NAD(P) (Fig. 3*D*). Mutational analysis however showed that substituting Glu1482 with alanine had only a mild impact on toxicity, whereas the K1392A variant was attenuated (Fig. 3*E*). These results suggest that Lys1392 makes a primary contribution, likely by electrostatic engagement of the pyrophosphate/ribose region or by stabilizing a reaction intermediate, while Glu1482 is not critical for catalytic output. The non-essential role of the canonical acidic residue and the modest effects of the lysine residue mutation indicate that the predicted catalytic pocket does not correspond to a *bona fide* ART active site. This apparent mismatch between fold-based predictions and catalytic requirements is however in agreement with the functional assays. Although the Tne5 domain adopts an ART-like architecture, it fails to catalyze protein ADP-ribosylation (Fig. S4, *A* and *B*) but instead efficiently depletes intracellular NAD *in vivo* and consumes NADH and NADPH *in vitro* (Figs. 4, *A* and *B* and S5). Moreover, NMR analysis identified nicotinamide and ADP-ribose as reaction products of NADH cleavage (Figs. 4*C* and S6). These observations suggest that Tne5 does not operate as a classical ART but rather as an NAD-consuming enzyme, such as an NADase, or as a highly divergent ART in which water, rather than a protein or RNA substrate, acts as the ADP-ribose acceptor. The identification of Tne5 as a distinct clade substantially extends the known diversity of T6SS-associated NAD(P)-consuming effectors with ART-like folds, reinforcing the notion that ART-like scaffolds provide a versatile platform that can be repurposed toward fundamentally different catalytic outcomes, ranging from macromolecule modification to cofactor hydrolysis. In this context, the predicted Lys1392-Glu1482 pocket may primarily contribute to NAD(P) binding or positioning without defining a canonical transferase active center. Furthermore, the comparable hydrolysis activity of NAD^+^ and NADPH suggests relaxed dinucleotide specificity of the active site, which is compatible with the geometry of the modeled pocket and with the modest contribution inferred for Glu1482. Further mutagenesis studies would be required to further define the catalytic mechanism of Tne5.

Taken together, these results demonstrate that Tne5 represents a new addition to the growing repertoire of NAD(P)-targeting enzymes, which are highly potent trans-kingdom toxins as NAD(P) cofactors play an essential and conserved role in the metabolism of all living organisms. Consistent with this, Tne5-like domains are broadly distributed within T6SS, contact-dependent inhibition, and type VII secretion systems, and more distantly related Tne5-like domains were also detected fused to ShK modules in eukaryotic proteins from nematodes and sea anemones, (Fig. 5*B*), highlighting their modularity and evolutionary success as polymorphic toxins in a wide range of biological and ecological contexts. This mirrors recent observations for ADP-ribosyl cyclases (46, 47) and supports a unifying view in which NAD(P) depletion constitutes a central antibacterial strategy recurrently selected for delivery by distinct secretion platforms.

## Experimental procedures

### Bacterial strains, growth conditions, media and chemicals

Strains used in this study are listed in Table S1. *Pluralibacter gergoviae* ATCC33028 was kindly provided by A. Davin-Régli and J.M. Bolla (MCT, Marseille, France). *E. coli* strains DH5α and CC118λpir were used for cloning, whereas *E. coli* W3110 was used for toxicity and bacterial competition assays. *P. gergoviae* and *E. coli* cells were grown at 37 °C in Lysogeny Broth (LB) with agitation or on LB agar (1.5%) plates. When needed, media were supplemented with ampicillin (100 μg mL^−1^), chloramphenicol (30 μg mL^−1^), or streptomycin (100 μg mL^−1^). Gene expression was induced by the addition of 0.2% of L-arabinose or of 0.05 mM of IPTG, or repressed with 1% of glucose.

### Plasmid construction

Plasmids and oligonucleotides used in this study are listed in Table S1. All cloning procedures were performed using Sequence and Ligation Independent Cloning (SLIC). Oligonucleotides were purchased from IDT. DNA fragments encompassing *tssL* or encoding Rhs^Pg^-CT or the Tni5 immunity were amplified from *P*. *gergoviae* genomic DNA using the Q5 polymerase (NEB). Vector backbones and full plasmid templates were amplified using the Q5 polymerase (NEB). PCR products were purified using NucleoSpin Gel and PCR Clean-up columns (Macherey-Nagel), digested with the appropriate restriction enzymes (NEB), and repurified prior to ligation overnight at 16 °C with T4 DNA ligase (NEB). Ligated plasmids were transformed into *E. coli* DH5α or CC118λpir and screened by colony PCR using EconoTaq PLUS GREEN 2 × Master Mix (Biosearch Technologies). Plasmid DNA was extracted using the NucleoSpin Plasmid kit (Macherey-Nagel) and verified by Sanger sequencing (Eurofins). Constructs were selected on LB agar plates containing ampicillin (for pNDM220), chloramphenicol (for pBAD33), or streptomycin (for pKNG101). Glucose (1%) was added for selection with the pNDM220 vector. Site-directed mutagenesis of *rhs*^*Pg*^*-CT* was performed using SLIC primers carrying the desired codon mutations for a PCR reaction amplifying the whole plasmid. After treatment with T4 DNA polymerase, plasmids were annealed and transformed into DH5α competent cells. All plasmids were checked by colony-PCR and verified by DNA sequencing (Eurofins).

### *Construction of the isogenic* tssL *and* rhs^Pg^-CT-imm *mutants of* P. gergoviae

The *P. gergoviae* isogenic *tssL* mutant strain, consisting at the replacement of codons seven and eight by two stop codons, was constructed as follows. Two 600-bp fragments corresponding to the sequences upstream and downstream of the codons to be mutated were generated by PCR using *P. gergoviae* chromosomal DNA as template, the Q5 DNA polymerase and primers pKNG-TssL-SLIC-1 and TssL-STOPSTOP-SLIC-2, and TssL-STOPSTOP-SLIC-3 and TssL-pKNG-SLIC-4, respectively. These two PCR products overlap on a 20-bp region and are centered on the mutated codons. Both fragments were cloned by SLIC into the suicide vector pKNG101 digested with BamHI using the T4 DNA polymerase, yielding pKNG101-tssLSTOP. The Δ*rhs*^*Pg*^*-CT-imm* mutant strain, consisting at the introduction of a stop codon at position 1330 in the *rhs* gene and the deletion of the sequence corresponding to the Tne5 domain and of the downstream open reading frame (encoding the Tni5 immunity protein) was constructed similarly. Two 900-bp fragments corresponding to the sequences upstream and downstream of the fragment to be deleted were generated by PCR using *P. gergoviae* chromosomal DNA as template, the Q5 DNA polymerase and primers pKNG-del-ToxImm-Pg-SLIC-1 and del-ToxImm-Pg-SLIC-2, and del-ToxImm-Pg-SLIC-3 and del-ToxImm-Pg-pKNG-SLIC-4, respectively. Both fragments were cloned by SLIC into pKNG101 digested with BamHI using the T4 DNA polymerase, yielding pKNG101-del-ToxImm. pKNG101-tssLSTOP and pKNG101-del-ToxImm were introduced into *P. gergoviae* by conjugation using the MFDpir conjugative strain. The first event of homologous recombination was selected on LB-agar plate supplemented with streptomycin. Several transconjugants were streaked onto LB agar without NaCl supplemented with 6% of sucrose to select the second recombination event. After incubation for 48 h at 12 °C, plates were incubated ON at 37 °C. Several clones were then tested for streptomycin sensitivity and checked by colony-PCR.

### Interbacterial competition assay

Antibacterial activity was measured using the CPRG-based lysis-associated β-galactosidase assay as previously described (61). Briefly, this assay is based on the degradation of CPRG, a membrane-impermeable chromogenic substrate of the β-galactosidase, into chlorophenol-red, by the β-galactosidase released by the lysis of *E. coli* W3110 *lacZ*^*+*^ recipient cells. Attacker *P. gergoviae* (WT, *tssL*^stop^ or Δ*rhs*^*Pg*^*-CT-imm)* and recipient *E. coli* W3110 strains were grown overnight in LB medium, normalized to an optical density at λ = 600 nm (*A*_600_) of 1, and mixed to a 1:2 (attacker:target) ratio. 12 μl of the mixtures were spotted onto LB agar plates supplemented with 0.1 mM of IPTG. Plates were incubated for 4 h at 37 °C, and recipient cell lysis was observed by the addition of a 10-μl drop of 2 mM of CPRG (Roche) on the top of each spot. The data shown are representative of at least three independent experiments.

### Toxicity assay in *E. coli*

*E. coli* DH5α cells were co-transformed with the pNDM220 and pBAD33 plasmid pairs encoding either WT and mutant Rhs^Pg^-CT (Tne5), and Rhs^Pg^ immunity protein (Tni5), respectively, or empty vectors as controls. Transformants were selected on LB agar plates supplemented with ampicillin, chloramphenicol, and 1% of glucose. Overnight cultures grown in the presence of antibiotics and glucose were normalized to an *A*_600_ of 1, diluted in 10-fold series, and 10-μl drops were spotted on LB agar plates containing antibiotics and either 1% glucose (repression conditions) or 0.05 mM IPTG (toxin induction from pNDM220) and 0.2% L-arabinose (immunity induction from pBAD33). Plates were incubated at 37 °C for 16 to 20 h before imaging. The data shown are representative of at least three independent experiments.

### *In vitro* coupled transcription-translation assay

Coupled *in vitro* transcription-translation assays were performed as previously described (38, 39) using the PURExpressⓇ *In vitro* Protein synthesis kit (NEB). Tne5 was obtained using a DNA template amplified using the 5’UTR-Tne5Pg and 3′UTR-Tne5Pg-strep primers. Five-μl IVT reactions typically yielded 6 to 7 μm of Tne5. For testing the impact of Tne5 on transcription/translation of the GFP, a second coupled *in vitro* transcription-translation reaction was then performed with a DNA template coding for the GFP-strep reporter protein, amplified using the 5′UTR-GFP and 3′UTR-GFP-strep primers. When indicated, reactions contained 0.1 mM NAD^+^ and/or 1.3 μm of IVT-produced Tne5. Reactions were performed for 2 h at 37 °C, proteins were separated by SDS-PAGE, transferred onto nitrocellulose membranes, and GFP-strep and Tne5-strep were detected by immunoblotting with strep-Tag antibodies (Classic, BioRad). The data shown are representative of three independent experiments.

### Ex-vivo *biotin-NAD protein labeling assay*

Fifty mL of *E. coli* DH5α cells were grown to late exponential phase and resuspended in 0.5 ml of Phosphate-buffered saline (PBS) buffer. Cells were then disrupted by sonication and unbroken cells were discarded. After addition of 0.1 mM of 6-biotin-17-NAD^+^ (Biolog Life Science) and of 0.2 μm of IVT-produced Tne5 to 30 μl of cell lysate, the mixture was incubated for 30 min at 25 °C. An equal volume of 2 × Laemmli loading dye was added, heated for 5 min at 95 °C, prior to protein separation by SDS-PAGE, transfer onto nitrocellulose membranes and immunodetection of biotin-ADP-ribosylated proteins with streptavidin-alkaline phosphatase conjugate (Invitrogen). The data shown are representative of three independent experiments.

### Intracellular NAD measurements

*E. coli* DH5α cells carrying the empty pNDM220 vector, or the pNDB220 vector carrying WT Tne5 or its K1392A or E1482A variants, or both pNDM220-Tne5 and pBAD33-Tni5 vectors were grown in LB medium supplemented with antibiotics and 1% of glucose to an *A*_600_ of 0.5. Cells were then washed in LB medium and resuspended in LB supplemented with 0.05 mM IPTG and 0.2% L-arabinose. After 1 h of induction at 37 °C, intracellular NAD levels were then quantified using a luminescence-based assay, by mixing one *A*_600_ unit of cells with the NAD/NADH-Glo detection reagent (Promega). NAD levels were then measured after 30 min of incubation at RT by recording luminescence using a TECAN microplate reader. The assays were performed from three independent cultures, each with technical triplicate, and data were analyzed using a one-way ANOVA test performed with the GraphPad Prism software (http://www.graphpad.com).

### *In vitro* NAD(P) degradation assay

Reactions were performed in 100 μl of PBS supplemented with 0.6 mM of NAD^+^ or of NADPH (Roche). After addition of IVT-produced WT and mutant Tne5 domains at the final concentration of 0.07 μm, samples were incubated at room temperature for 30 min. The reaction was then quenched by adding 50 μl of 6 M NaOH. Fluorescence was measured using a TECAN microplate reader (excitation: 340 nm; emission: 420 nm) after 30 min of incubation at room temperature in the dark. Controls including the NAD-consuming ARC^tox^ domain (47) and the Tre23 ART domain (38). The assays were performed from three independent IVT reactions, each with technical triplicate, and data were analyzed using a one-way ANOVA test performed with the GraphPad Prism software.

### Nuclear magnetic resonance (NMR) spectroscopy

Samples from 200-μl *in vitro* NAD ^+^ degradation reactions were supplemented with 10% of D_2_O (EurisoTop) and transferred into 3-mm, 7-inch thin-walled Pyrex NMR tubes (Innova-Chem). NMR spectra were acquired at 25 °C on a Bruker NEO 600 MHz spectrometer equipped with a cryogenically cooled triple-resonance TCI 5-mm probe (IMM NMR facility, Marseille). One-dimensional ^1^H NMR spectra were recorded for 125 s using a Watergate W5 pulse sequence for suppression of the solvent signal. For each spectrum, 64 transients were collected into 32 K time domain complex points with a spectral width of 16 ppm. Free induction decays were apodized using a shifted sine-bell window function prior to Fourier transformation. All ^1^H NMR spectra were manually phase-corrected using the TopSpin software (version 4.4.0, Bruker, Biospin, https://www.bruker.com/products-and-solutions/mr/nmr-softwares/topspin.html).

### Bioinformatics analyses and AlphaFold3 modeling

The T6SS locus was identified in the ATCC33028 genome assembly (GenBank accession: GCA_001598855.1) by BLASTP against canonical T6SS components. Domain architecture of the Rhs protein was annotated with HHpred and PFAM; transmembrane helices were predicted with TMHMM. Search for structural homologues in the PDB was conducted using the DALI server (62). Phylogeny reconstruction of the NADase-related proteins was performed using the IQ-TREE webserver (Los Alamos Laboratory) (63, 64). The maximum-likelihood tree was inferred using the VT+G4 substitution model, with branch support estimated from 1000 SH-aLRT replicates and 1000 Ultrafast bootstrap replicates (65), based on a multiple sequence alignment generated with Clustal Ω 1.2.4 (EMBL-EBI) using representative members of the Tne1-4 families, as well as previously characterized NAD(P) glycohydrolases, including *M. tuberculosis* Tuberculosis Necrotizing Toxin (59) and AfNADase (60), and the *P. ananatis* ADP-ribosyl cyclase (ARC^tox^, 47). The resulting tree was visualized and annotated using the Interactive Tree of Life (iTOL) server (66). Tne5 closest homologues were retrieved using JackHMMER (EMBL-EBI, (67))using a single iteration. AlphaFold3 model predictions were run on the AlphaFold server using default MSA generation (68). The toxin-immunity complex was predicted with AlphaFold3 multimer mode (stoichiometry 1:1). Models were visualized in ChimeraX. pLDDT and PAE as well as confidence metrics are indicated in the corresponding figures.

## Data availability

All data are contained within the article and the corresponding supporting information.

## Supporting information

This article contains supporting information (six Supplemental Figures and one Supplemental Table) .

## Conflict of interest

The authors declare that they do not have any conflicts of interest with the content of this article.

## Supporting information

Supplmentary Material
