# Supplementary material for: An ART-fold Rhs toxin from Pluralibacter gergoviae defines Tne5, a novel family of NAD(P) glycohydrolases effectors
Source: J Biol Chem. 2026 May 22;302(7):113182. doi: 10.1016/j.jbc.2026.113182 (PMC13285283; doi:10.1016/j.jbc.2026.113182)
Supplement: Supplmentary Material [file mmc1.pdf]

Supporting Information

An ART-fold Rhs toxin from *Pluralibacter gergoviae* defines Tne5, a novel clade of NAD(P) glycohydrolases effectors

Jonas B. Desjardins, Martin Durrmeyer, Olivier Bornet, Eric Cascales

Table S1. Strains, plasmids and oligonucleotides used in this study.

**STRAINS**		
**Strains**	**Description**	**Source/Reference**
Pluralibacter gergoviae		
P. gergoviae ATCC 33028	Wild-type *Pluralibacter gergoviae* NBRC 105706 (ATCC 33028)	(69)
Pg-*tssL*^{stop}	insertion of two consecutive STOP codons after the START codon of *tssL* (A8H26_06215)	This study
Pg-Δ*rhs*^{Pg}-*CT-imm*	deletion of the *rhs*^{Pg} (A8H26_06345) sequence corresponding to the Tne5 toxin domain (codons 1330-1503) and *imm* gene (A8H26_06350)	This study
E. coli K-12		
DH5α	F^-, Δ*(argF-lacZ)*U169, *phoA, supE44,* Δ(lacZ)M15, *relA, endA, thi, hsdR*	New England Biolabs
CC118λpir	Δ*(ara-leu) araD* Δ*lacX galE galK phoA thi rpsE rpoB argE recA λpir* phage lysogen	(70)
W3110	F^-, λ^- *rph*1 INV(*rrnD*, *rrnE*)	Laboratory collection
MFDpir	*MG1655* RP4-2-Tc::[ΔMu1*::aac(3)IV* Δ*aphA* Δ*nic35* Δ*Mu2::zeo* Δ*dapA::(erm-pir)* Δ*recA*	(71)
**PLASMIDS**		
pNDM220 medRBS	Mini-R1, single-copy vector, LaqI^q , $P_{A1/04/03}$, with an attenuated RBS, Amp^R	Laboratory collection
pNDM-Tne5^{Pg}	*P. gergoviae tne5* (Rhs amino acids 1330–1503) cloned into pNDM220 medRBS	This study
pNDM-Tne5^{Pg} K1392A	Lys1392 to Ala substitution in pNDM-Tne$_{Pg}$	This study
pNDM-Tne5^{Pg} E1482A	Glu1482 to Ala substitution in pNDM-Tne$_{Pg}$	This study
pBAD33 RBS	Expression vector, AraC, arabinose-inducible, Ribosome binding-site, Cm^R	Laboratory collection
pBAD-Tni5^{Pg}	*P. gergoviae tni5* (A8H26_06350) cloned into pBAD33 RBS	This study
pKNG101	R6K origin, TRK2 origin, mobRK2, *sacB*$^+$, Sm^R	(72)
pKNG101-*tssL*STOP	pKNG101 bearing the regions flanking the *tssL* codon 7 and inserting two STOP codons	This study
pKNG101-delTox-Imm	pKNG101 bearing the regions flanking the *rhs*^{Pg}-CT-coding sequence and *imm* gene	This study
**OLIGONUCLEOTIDES**		
Construction of *P. gergoviae* mutants [a]		

pKNG-TssL-SLIC-1	CCCTGCAGGTCGACGGATCCCGAATTTACCCTGCTGACGC
TssL-STOPSTOP-SLIC-2	AGCCTGCTGTTGCTGCATAG
TssL-STOPSTOP-SLIC-3	CTATGCAGCAACAGCAGGCTTAGTAAAGCACGCAGGAACAACAGGC
TssL-pKNG-SLIC-4	CTTATGGTACCCGGGGATCCTGGTAGATTTTCGCCAGCACC
Verif-TssL-STOPSTOP	AGCAACAGCAGGCTTAGTAA
pKNG-del-ToxImm-Pg-SLIC-1	CCCTGCAGGTCGACGGATCCCGCTGGGACTACGACTACCG
del-ToxImm-Pg-SLIC-2	CAACCCCCACGGATCCACCC
del-ToxImm-Pg-SLIC-3	GGGTGGATCCGTGGGGGTTGTAACGTTATCAACGAGCGAAAGTTTATGTTG
del-ToxImm-Pg-pKNG-SLIC-4	CTTATGGTACCCGGGGATCCTACCGTGAACATGGTCTGCC
5-Verif-del-ToxImm	GGGGCTGCTCACCAGCC
3-Verif-del-ToxImm	GACTGGCGGATGAACTTCCGG
Plasmid construction [b,c]	
5-pNDMmedrbs-SpeI-Tne5^{Pg}	GATCACTAGT**ATG**AGTTGTAAGAATAGTTGGAATGAATTTCAGAGTAG
3-pNDMmedrbs-EcoRI-Tne5^{Pg}	GATCGAATTCTCATATATTACTATTTGGCAATTTAGATATCACTGCCTCC
5-pBAD33-SalI-Tni5^{Pg}	GATCGTCGACATGAAAGAGGTTATATTTAGTAAGTACGGTATAGATATTC
3-pBAD33-HindIII-Tni5^{Pg}	GATCAAGCTTTTATTTAATATAAGGCTTATATCCATTATCTCTATTCTGAGACGC
Site-directed mutagenesis [d]	
Tne5$_{Pg}$-K1392A-SLIC1	AGATACTCCACCATCAAATTGCGC
Tne5$_{Pg}$-K1392A-SLIC2	TTGATGGTGGAGTATCTGCGATTGCATGGGGAGTTAATCGTGC
Tne5$_{Pg}$-E1482A-SLIC1	CGTCACCGGTGTTTGGTCAATAAC
Tne5$_{Pg}$-E1482A-SLIC2	ACCAAACACCGGTGACGGCGGCAGTGATATCTAAATTGCCAAATAG
DNA fragments for *in vitro* transcription-translation [e]	
5'UTR-GFP	GCGAATTAATACGACTCACTATAGGGCTTAAGTATAAGGAGGAAAAAATATGAGTAAAGGAGAAGAACTTTTCAC
3'UTR-GFP-strep	AAACCCCTCCGTTTAGAGAGGGGTTATGCTAGTTATTATTTTTCGAACTGCGGGTGGCTCCATTTGTATAGTTCATCCATGCCA
5’UTR-Tne5^{Pg}	GCGAATTAATACGACTCACTATAGGGCTTAAGTATAAGGAGGAAAAAATATGAGTTGTAAGAATAGTTGGAATGAATTTCAG

3’UTR-Tne5^{Pg}-strep

AAACCCCTCCGTTTAGAGAGGGGTTATGCTAGTTATTA<u>TTTTTCGAACTGCGGGTGGCTCCA</u>TATATTACTATTTGGCAATTTAGATATCAC

[a] sequence complementary to the plasmid (SLIC) underlined.

[b] restriction site underlined.

[c] ATG sequence added in bold.

[d] mutagenized codon underlined.

[e] Streptag sequence underlined

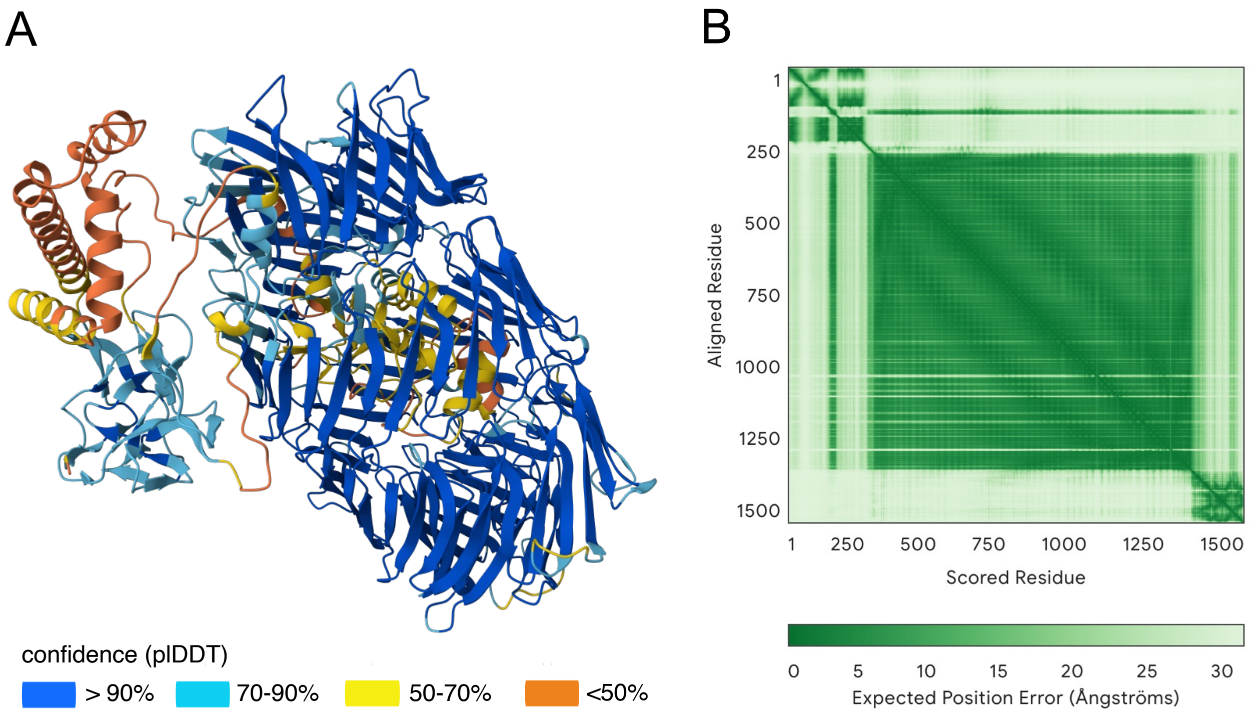

Supplemental Figure 1. AlphaFold3 structural model of full-length Rhs^{Pg}. *A*, Structural model of Rhs^{Pg} colored by predicted Local Distance Difference Test (pLDDT) scores, ranging from blue (high confidence, >90) to orange (low confidence, <50). *B*, Predicted Aligned Error (PAE) plot of Rhs^{Pg} showing the expected position error (in Å) for each residue pair (dark green, high confidence; light green, low confidence).

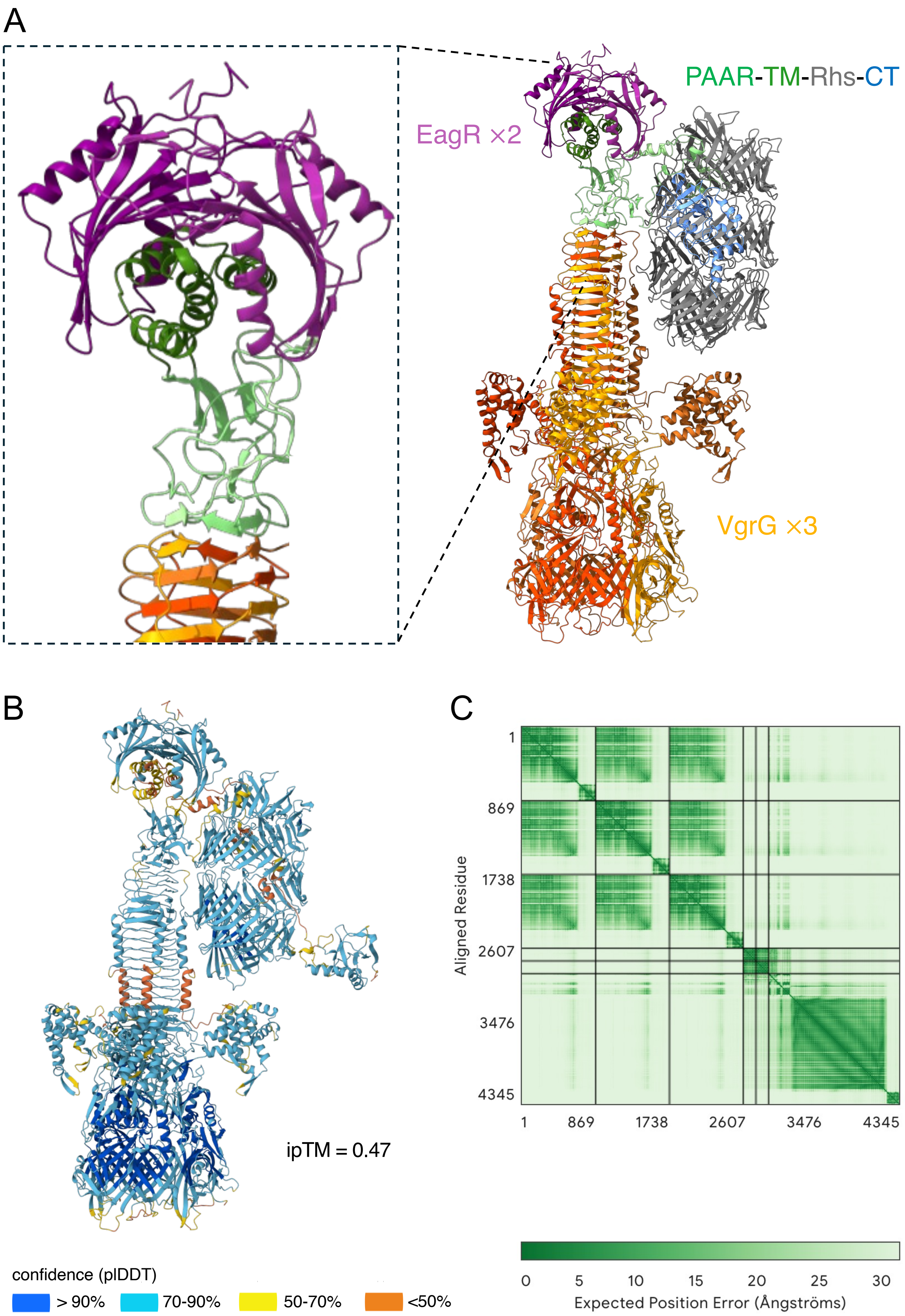
A
PAAR-TM-Rhs-CT
EagR ×2
VgrG ×3
B
ipTM = 0.47
confidence (plDDT)
> 90%
70-90%
50-70%
<50%
C
Aligned Residue
1
869
1738
2607
3476
4345
0
5
10
15
20
25
30
Expected Position Error (Ångströms)

Supplemental Figure 2. Predicted assembly of the Rhs^{Pg} effector with VgrG and the cognate EagR-like chaperone. *A*, AlphaFold3 multimer model of the effector-loaded spike complex including a trimer of VgrG (shades of orange), a dimer of EagR chaperones (purple), and the Rhs^{Pg} effector. The different Rhs^{Pg} domains are colored as in Fig. 1*C-D* (PAAR domain, light green; TMD, dark green; Rhs core, grey; C-terminal extension, blue). A close-up view of the complex, emphasizing the interface between the VgrG tip and the Rhs^{Pg} PAAR domain, as well as the protection of the TMD by the EagR dimer, is shown in the inset on left. *B*, AlphaFold3 multimer model of the effector-loaded spike complex colored by pLDDT confidence scores. The overall interfacial confidence score (ipTM, 0.47) is indicated. ipTM scores of VgrG-PAAR and EagR-TMD subcomplexes are 0.66 and 0.8, respectively. *C*, Predicted aligned error (PAE) plot of the effector-loaded spike complex.

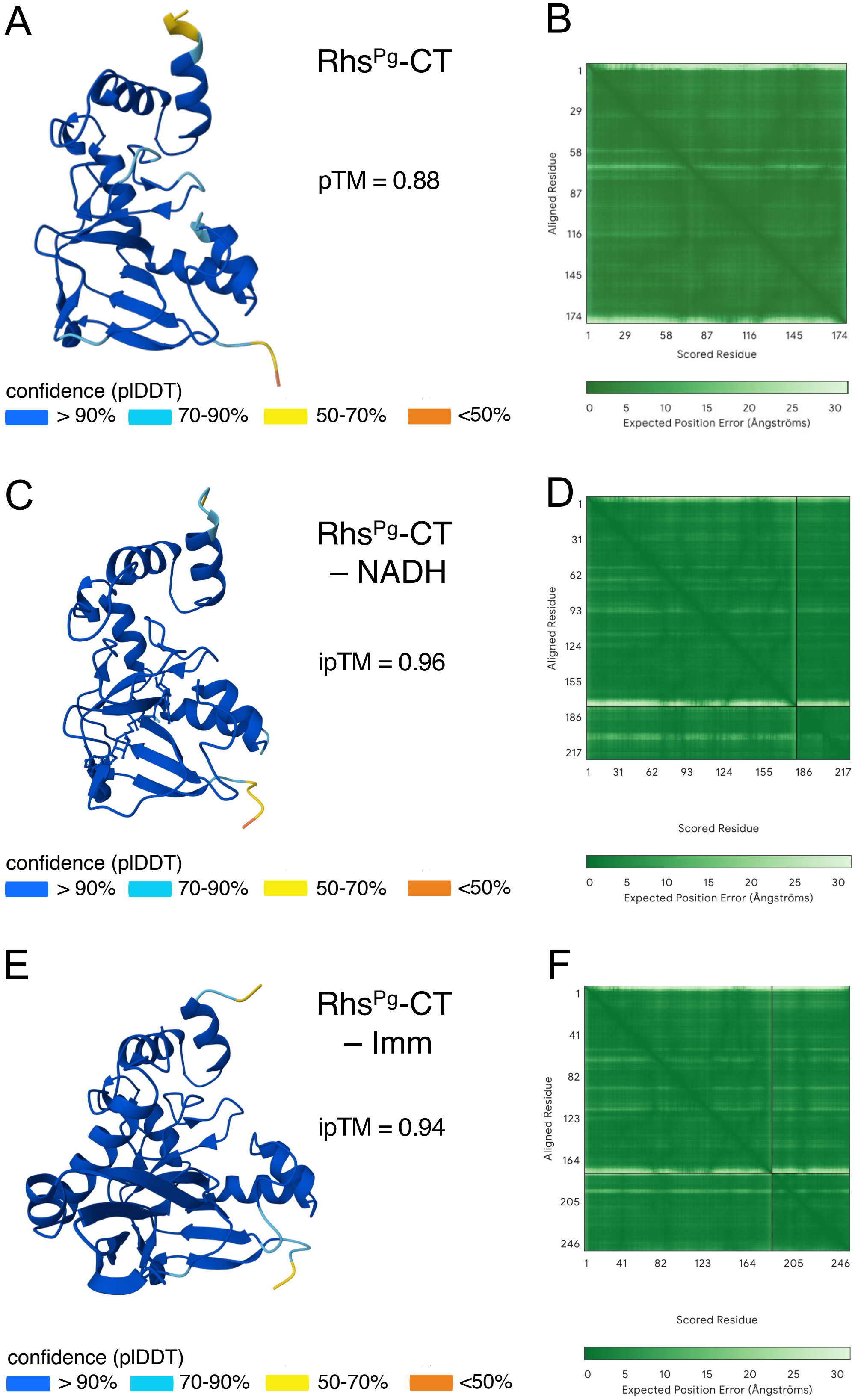

Supplemental Figure 3. AlphaFold3 structural models of the Rhs^{Pg} C-terminal toxin domain alone, or in complex with ligand or immunity. (*A*, *C*, *E*) Predicted structures of the Rhs^{Pg}-CT domain alone (*A*), bound to NADH (*C*), or in complex with its cognate immunity protein (*E*) colored by pLDDT. Confidence scores (pTM or piTM) are indicated. (*B*, *D*, *F*) PAE plots of the Rhs^{Pg}-CT domain alone (*B*), bound to NADH (*D*), or in complex with its cognate immunity protein (*F*).

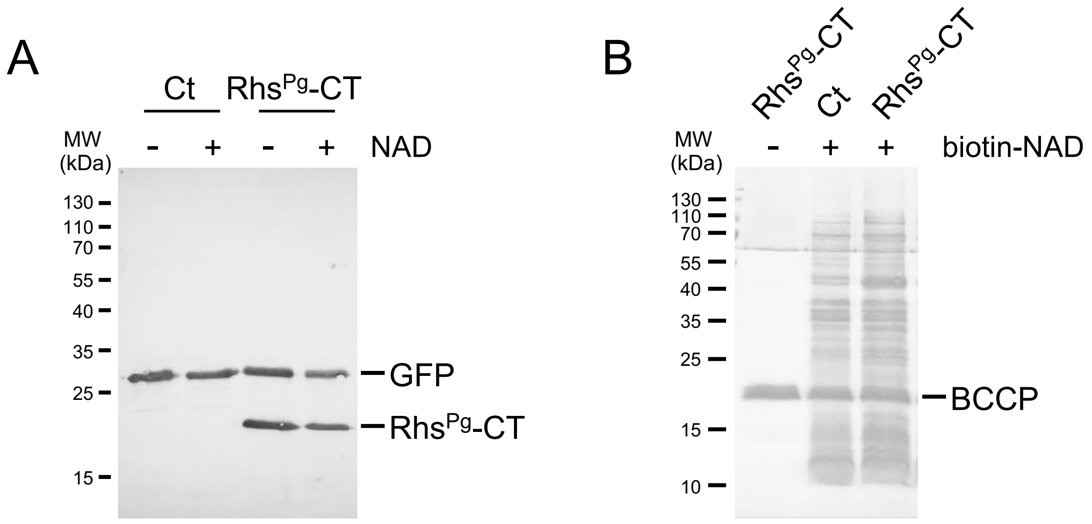

Supplemental Figure 4. Rhs^{Pg}-CT has no measurable effect on translation or protein ADP-ribosylation. *A,* Coupled *in vitro* transcription-translation. *In vitro* transcription-translation of the Strep-tagged GFP reporter protein in reactions supplemented with NAD$^+$ and Strep-tagged Rhs^{Pg}-CT, as indicated. After 3 h of incubation, proteins were separated by SDS-PAGE, transferred onto nitrocellulose and immunodetected using anti-Streptag antibody. Molecular weight standards (MW, in kDa) are indicated on the left. *B, Ex vivo* protein ADP-ribosylation assays of cellular extracts of *E. coli* in the presence of Rhs^{Pg}-CT and of biotin-labelled NAD$^+$ (biotin-NAD), as indicated. Proteins were separated by SDS-PAGE, transferred onto nitrocellulose and biotinylated proteins were detected using Streptavidin-Alkaline Phosphatase conjugate. Molecular weight standards (MW, in kDa) are indicated on the left. The only naturally biotinylated protein of *E. coli* (Biotin carboxyl carrier protein, BCCP) is indicated on the right.

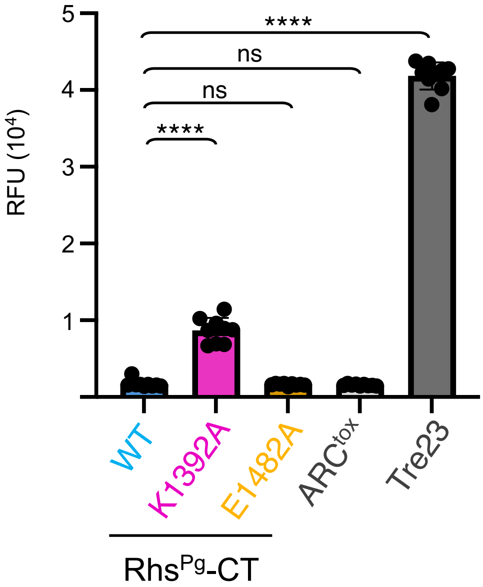

Supplemental Figure 5. Rhs^{Pg}-CT displays NADPH glycohydrolase activity *in vitro.* NADPH glycohydrolase assay. Fluorescence-based assay measuring NADPH levels after incubation of 0.6 mM of NADPH with 0.07 µM of wild-type (WT) Rhs^{Pg}-CT (blue bar) or its K1392A (purple bar) or E1482A (orange bar) variants for 30 min. Control reactions with 0.07 µM of ARC^{tox} (NAD(P)-consuming, light grey bar) or of Tre23 (ART, dark grey bar) were included. RFU, relative fluorescence units. Bars represent the mean of nine independent reactions (each independent value indicated by circles). Standard deviations are indicated. **** denotes a statistically significant difference between the conditions (one-way ANOVA; p<0.0001; ns, non-significant).

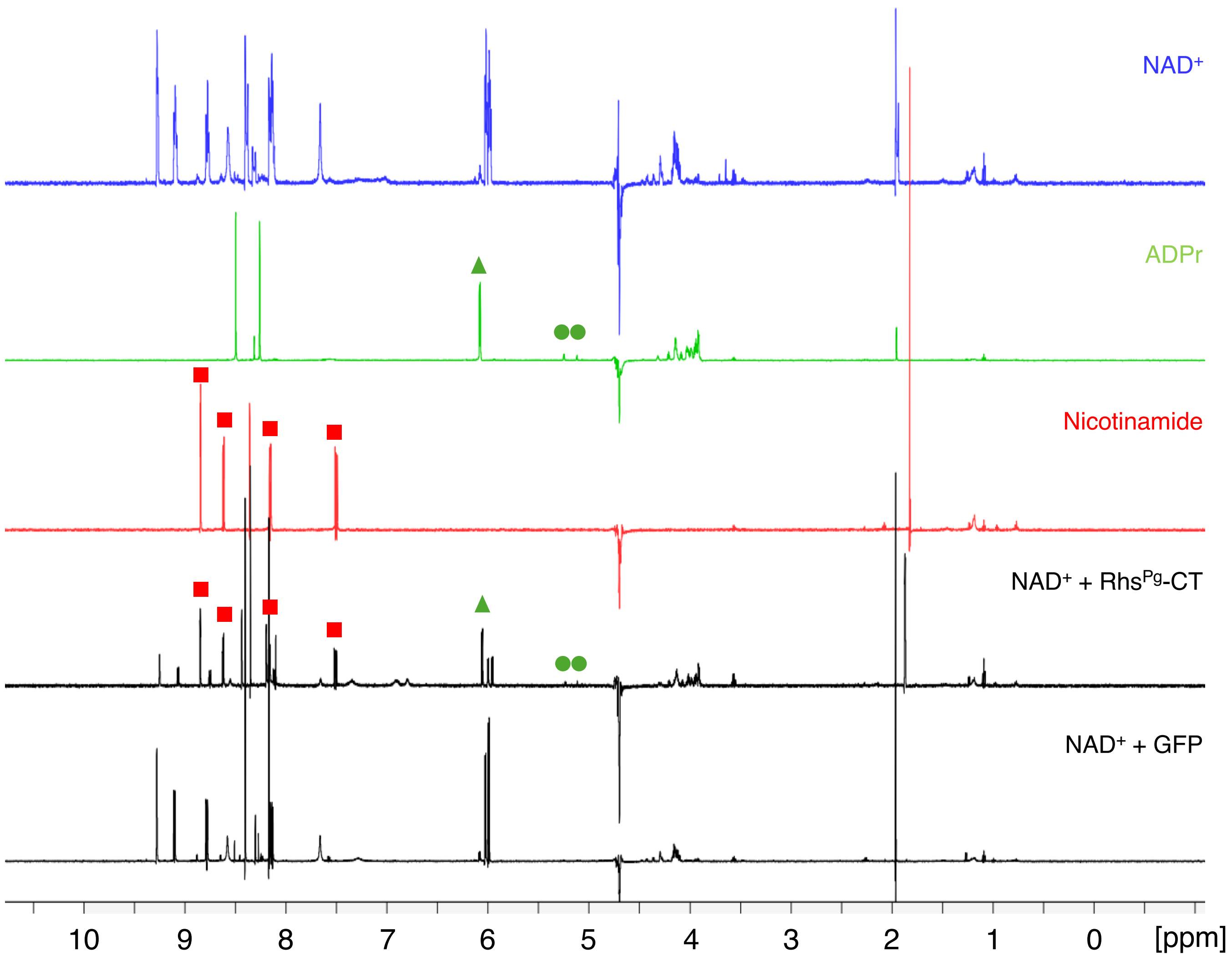

Supplemental Figure 6. Rhs^{Pg}-CT hydrolyzes NAD^{+} into nicotinamide and ADP-ribose. One-dimensional ^{1}H NMR spectra of reference compounds (NAD^{+}, blue; ADP-ribose, green; nicotinamide, red), and of reaction mixtures obtained after 30-min incubation of NAD^{+} in the presence of 0.07 µM of GFP or of Rhs^{Pg}-CT, recorded at 600 MHz. In the ADP-ribose spectrum (green), the signal at 6.1 ppm (green triangle) corresponds to the H1' proton of the ADP moiety, while the two signals at 5.2 and 5.1 ppm (green circles) correspond to the anomeric H1' protons of the ribose moiety in the absence of the nicotinamide group. In the nicotinamide spectrum (red), peaks at 8.8, 8.6, 8.15 and 7.5 ppm (red squares) correspond to the four aromatic protons of the nicotinamide ring (73). These spectral features are also observed in the reaction mixture containing Rhs^{Pg}-CT but not in the GFP control.

Supplemental References

69. Brenner, D.J., Richard, C., Steigerwalt, A.G., Asbury, M.A., and Mandel, M. (1980) *Enterobacter gergoviae* sp. nov.: a new species of Enterobacteriaceae found in clinical specimens and the environment. *Int. J. Syst. Bacteriol.* **30,** 1–6.

70. Herrero, M., de Lorenzo, V., and Timmis, K.N. (1990) Transposon vectors containing non-antibiotic resistance selection markers for cloning and stable chromosomal insertion of foreign genes in gram-negative bacteria. *J Bacteriol.* **172,** 6557–6567.

71. Ferrières, L., Hémery, G., Nham, T., Guérout, A.M., Mazel, D., Beloin, C., and Ghigo, J.M. (2010) Silent mischief: bacteriophage Mu insertions contaminate products of *Escherichia coli* random mutagenesis performed using suicidal transposon delivery plasmids mobilized by broad-host-range RP4 conjugative machinery. *J Bacteriol.* **192,** 6418–6427.

72. Kaniga, K., Delor, I., and Cornelis, G.R. (1991) A wide-host-range suicide vector for improving reverse genetics in gram-negative bacteria: inactivation of the *blaA* gene of *Yersinia enterocolitica*. *Gene.* **109,** 137–141.

73. Shabalin, K., Nerinovski, K., Yakimov, A., Kulikova, V., Svetlova, M., Solovjeva, L., Khodorkovskiy, M., Gambaryan, S., Cunningham, R., Migaud, M.E., Ziegler, M., and Nikiforov, A. (2018) NAD metabolome analysis in human cells using ^{1}H NMR spectroscopy. *Int J Mol Sci.* **19,** 3906.
